# Success rate of an outreach PMTCT program in Nigeria

**DOI:** 10.1186/1471-2334-14-S2-P9

**Published:** 2014-05-23

**Authors:** EC Herbertson, AGI Ohihoin, TA Gbajabiamila, AN David, O Odubela, SO Ekama, CV Gab-Okafor, H Owhodo, OC Ezechi, IAO Ujah

**Affiliations:** 1 Nigerian Medical Institute of Research, Lagos, Nigeria

## Introduction

Prevention of mother-to child transmission (PMTCT) of HIV is an important strategy to achieving zero new HIV infection and has been an important strategy in preventing the upsurge of HIV infection in Nigeria.

## Method

This study is an 8 year (2004 – 2012) review to evaluate the outcome of PMTCT outreach services offered by the Nigerian Institute of Medical Research (NIMR). It is a retrospective review of PMTCT and infant follow-up databases. PMTCT enrollees (from the NIMR adult antiretroviral clinic and referrals from public and private antenatal clinics) were placed on routine PMTCT care and subsequent infant prophylaxis, based on the WHO/National PMTCT guidelines. While ANC services are provided at the clinic, labor and delivery services are provided by partner institutions. The women are referred out at 36 weeks with delivery protocol. They are referred back after delivery with complete delivery summary form. Infants were followed up for 18 months and had HIV DNA PCR done at 6 weeks and 6 months.

## Results

There were a total of 4139 records for PMTCT uptake. 257 of these had incomplete data and were excluded from analysis. The delivery outcomes include: miscarriage -107 (2.8%); still birth- 134 (3.5%); live births -3641 (94%), neonatal deaths-68 (1.8%).Only 2623(73%) of the live births were presented at the clinic for follow up; of these, 21(0.8%) tested HIV positive by DNA at an average age of 12 weeks.

## Conclusion

The PMTCT success rate of 99% achieved is comparable to that in developed countries. In low income countries where it is not possible to implement full PMTCT protocol, a strategy where a public institution is the center and provides services for other smaller institutions is recommended.

